# Supplementary material for: Enabling giant thermopower by heterostructure engineering of hydrated vanadium pentoxide for zinc ion thermal charging cells
Source: Nat Commun. 2023 Oct 26;14:6816. doi: 10.1038/s41467-023-42492-z (PMC10603064; doi:10.1038/s41467-023-42492-z)
Supplement: Supplementary file 1 — Supplementary Information [file 41467_2023_42492_MOESM1_ESM.pdf]

Supplementary Information

Enabling giant thermopower by heterostructure engineering of hydrated vanadium pentoxide for zinc ion thermal charging cells

Zhiwei Li[1], Yinghong Xu[1], Langyuan Wu[1], Jiaxin Cui[1], Hui Dou[1], Xiaogang Zhang[1,*]

[1]Jiangsu Key Laboratory of Electrochemical Energy Storage Technologies, College of Material Science and Technology, Nanjing University of Aeronautics and Astronautics, Nanjing 211106, China

[*]Correspondence to X. Z. (azhangxg@nuaa.edu.cn)

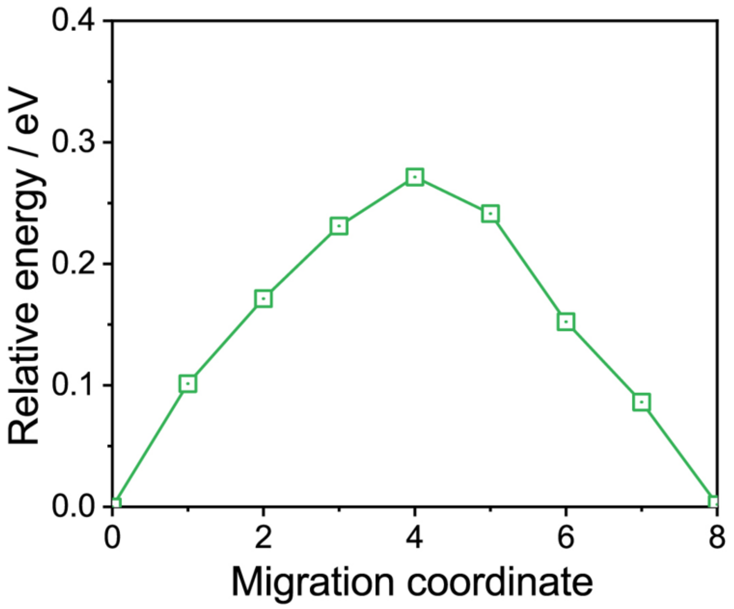

Supplementary Fig. 1 Energy barrier for ions diffusion in V_2O_5@rGO.

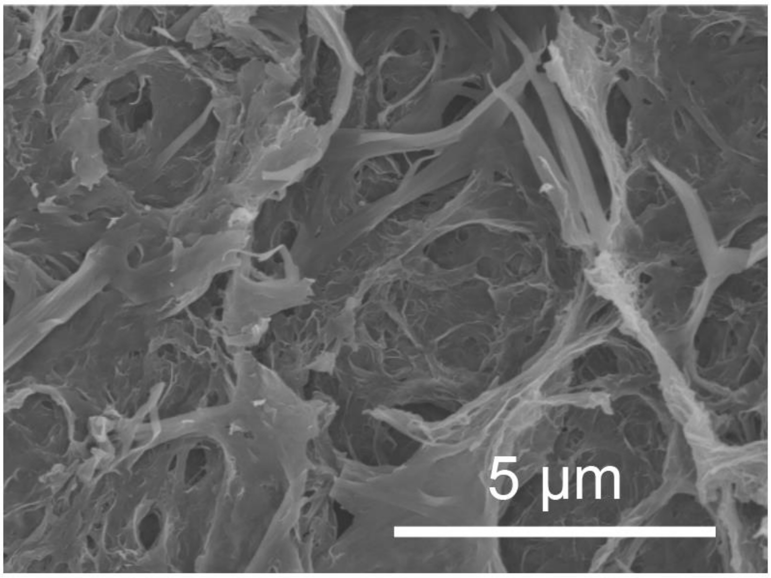

Supplementary Fig. 2 FESEM image of V_2O_5@rGO-1.5.

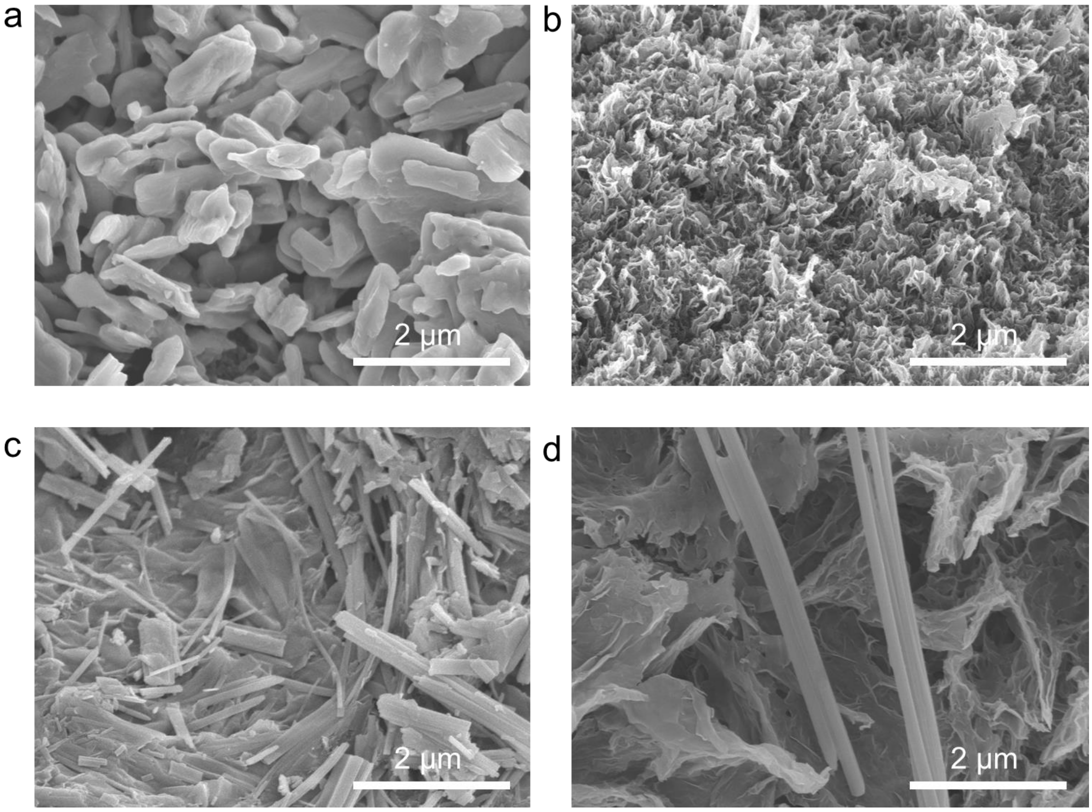

Supplementary Fig. 3 FESEM images of a V_2O_5, **b** rGO, **c** V_2O_5@rGO-1.0, and **d** V_2O_5@rGO-2.0.

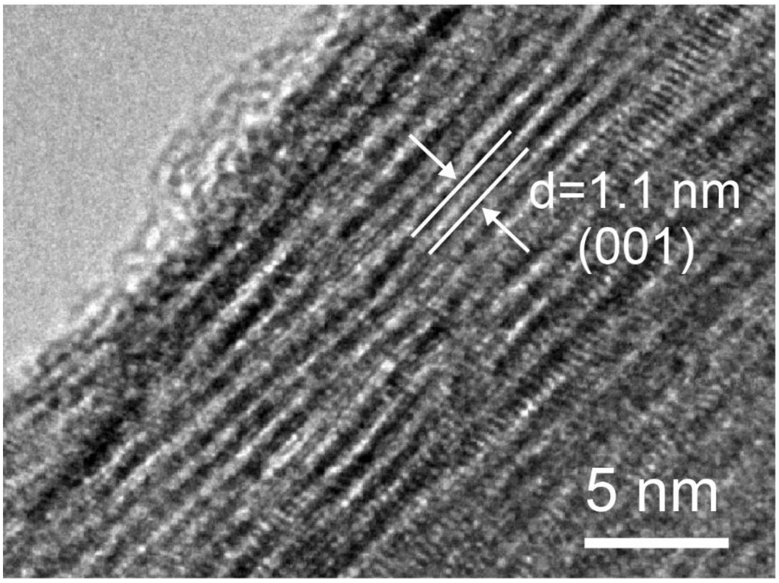

Supplementary Fig. 4 HRTEM image of V_2O_5@rGO-1.5.

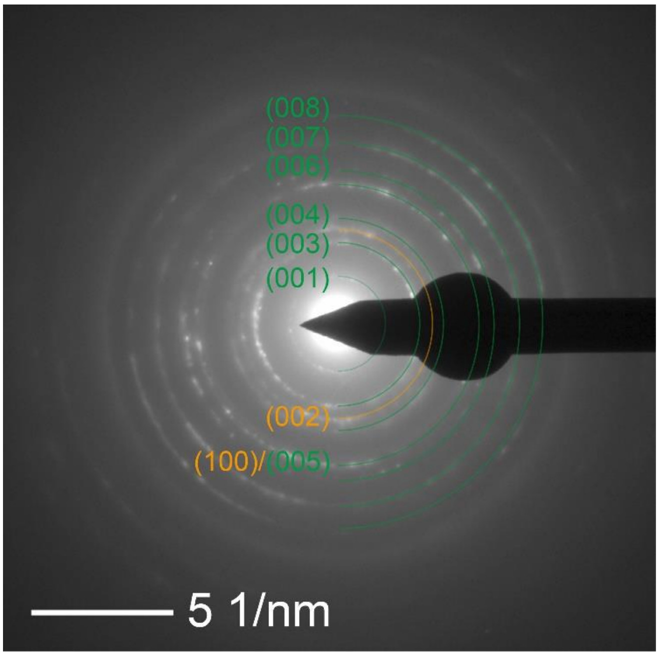

Supplementary Fig. 5 SAED pattern of V_2O_5@rGO-1.5.

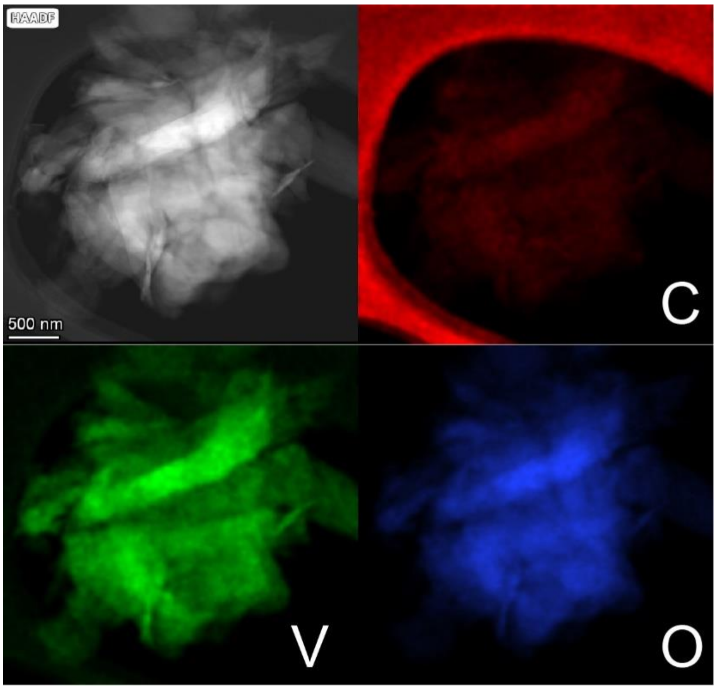

Supplementary Fig. 6 Elemental mapping images confirming the presence and distribution of C, V, and O elements in V_2O_5@rGO-1.5.

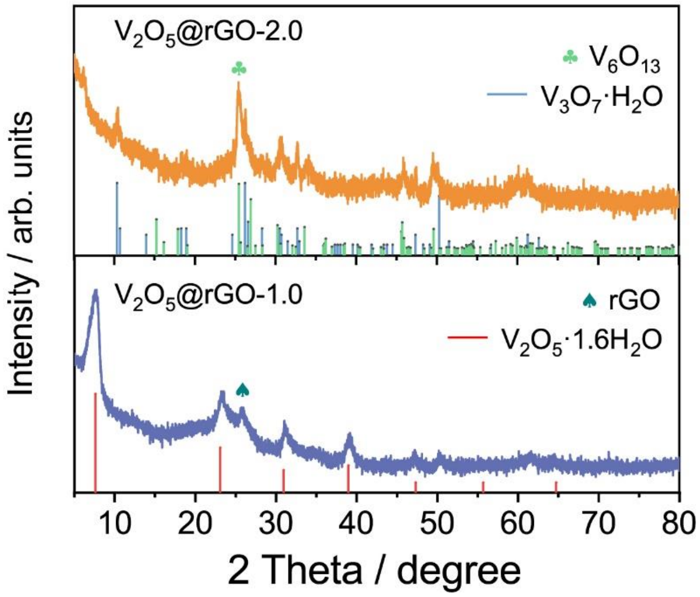

Supplementary Fig. 7 XRD pattern of V_2O_5@rGO-1.0, and V_2O_5@rGO-2.0.

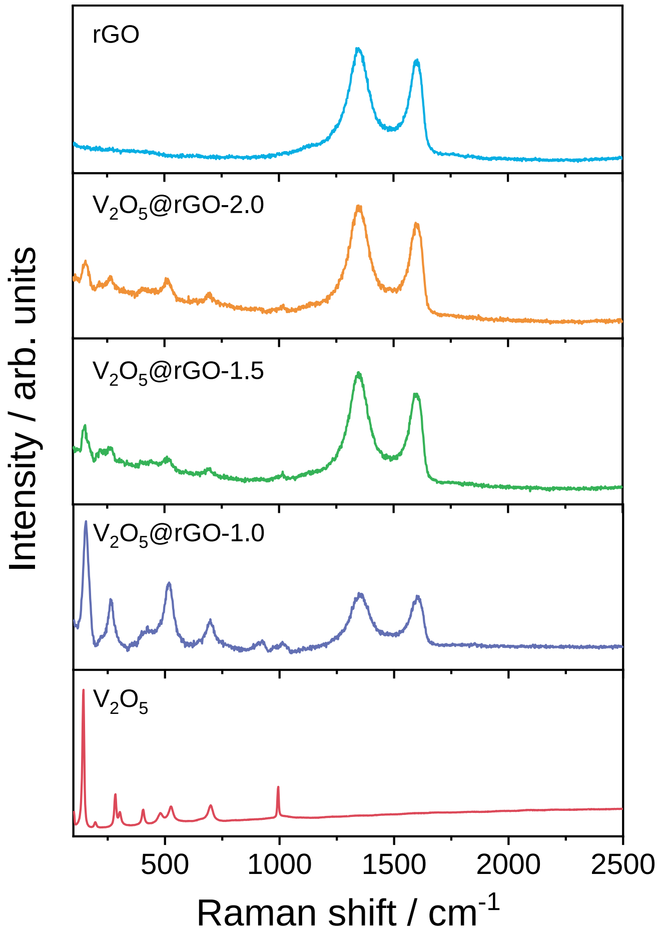

Supplementary Fig. 8 Raman spectra of various materials.

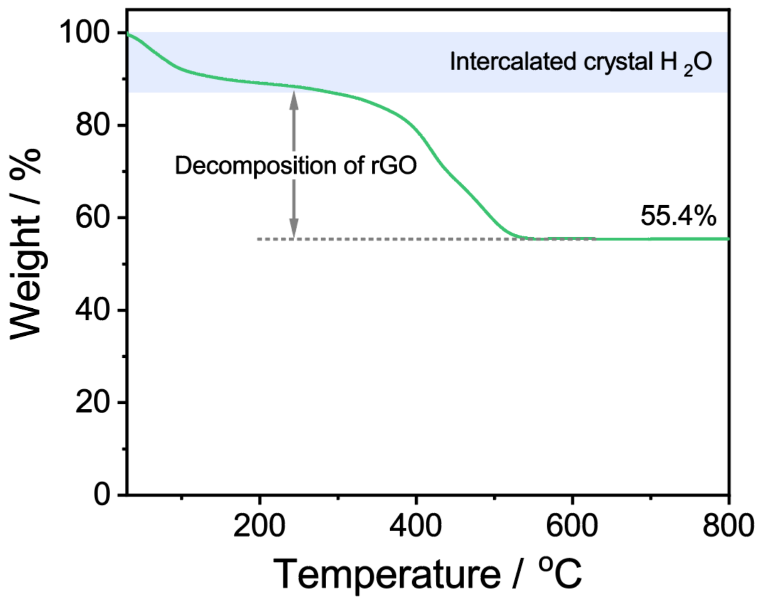

Supplementary Fig. 9 TGA curve of V_2O_5@rGO-1.5.

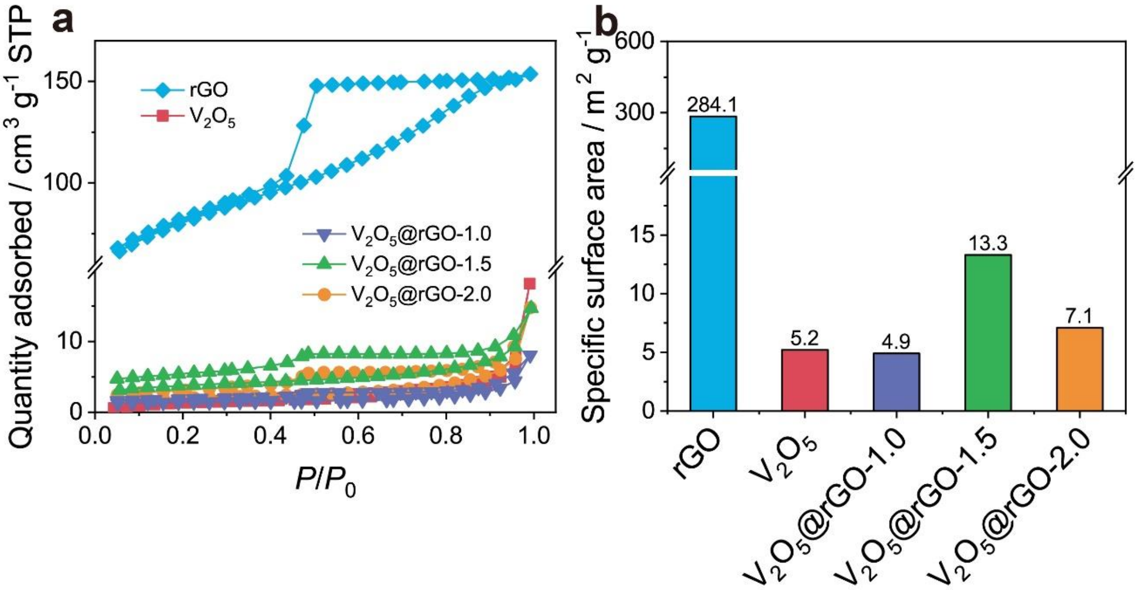

Supplementary Fig. 10 Porosity and surface area analyses for various samples. a N_2 adsorption-desorption isotherms, and **b** corresponding specific surface area values.

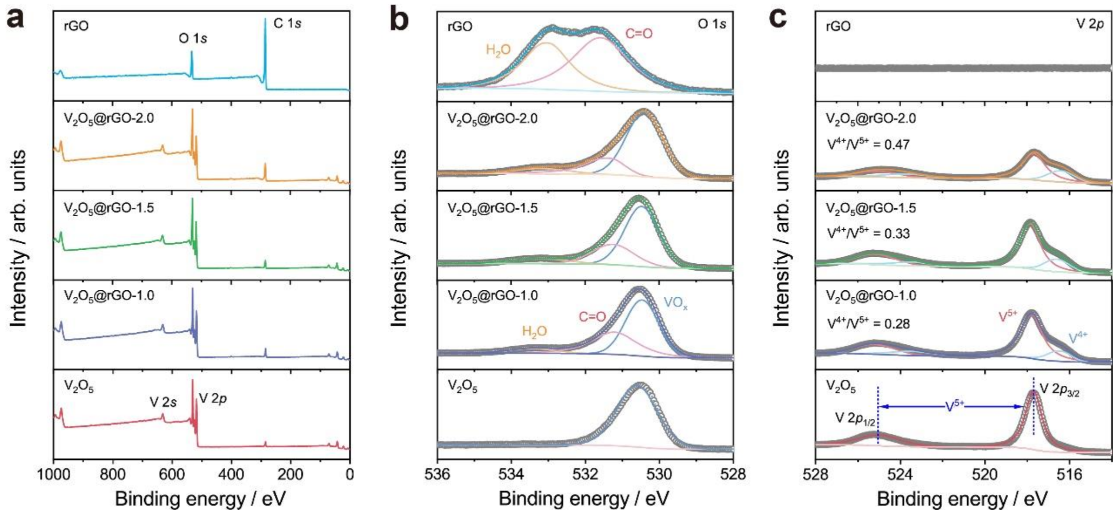

Supplementary Fig. 11 XPS analyses for various materials. a XPS survey. High-resolution XPS spectra of **b** O 1*s* and **c** V 2*p*.

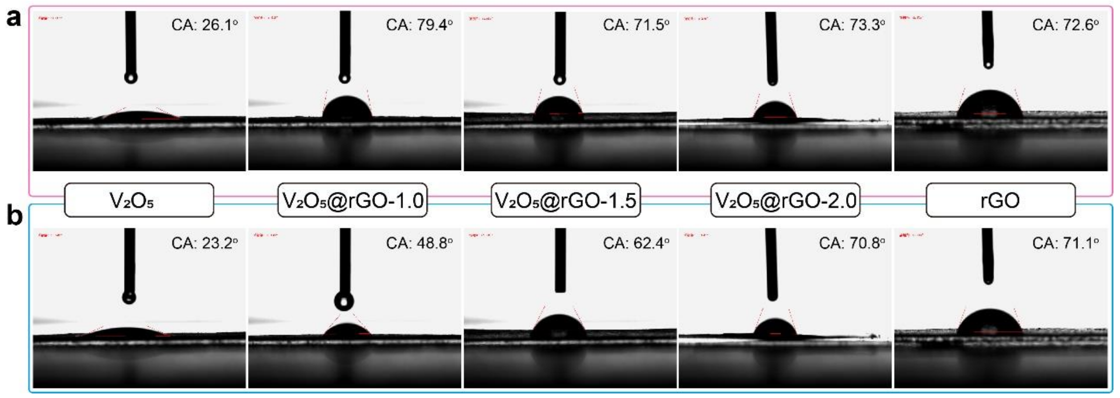

Supplementary Fig. 12 Contact angle analyses for films with various materials. **a** initial state, and **b** after standing 20 s.

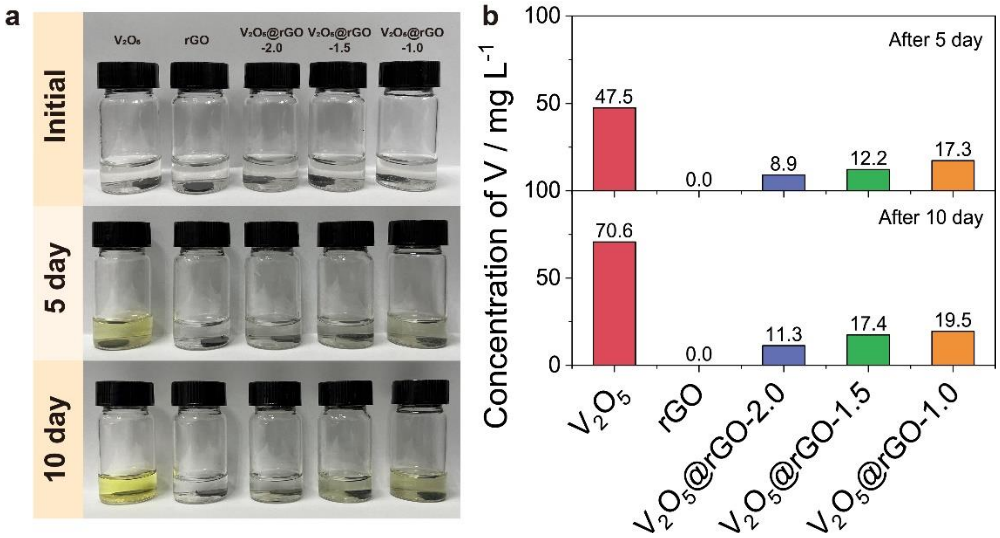

Supplementary Fig. 13 Dissolution of vanadium in 0.5 mol L^{-1} Zn(CF_3SO_3)$_2$ electrolyte with different soaking times. **a** digital photos. **b** ICP-OES results.

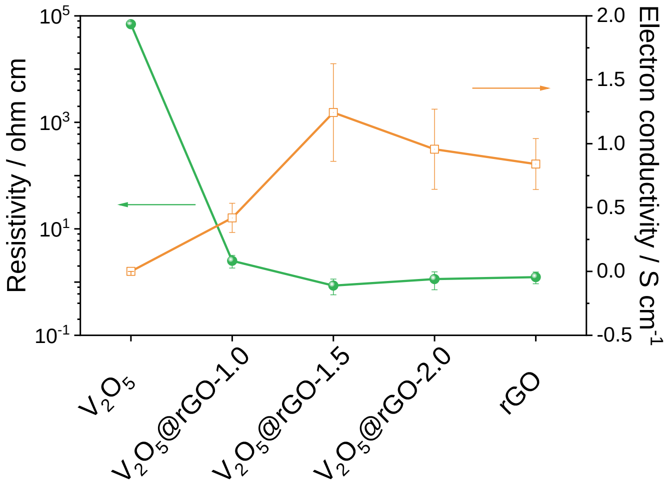

Supplementary Fig. 14 Resistivity and corresponding electron conductivity of various materials measured by four-point probe method.

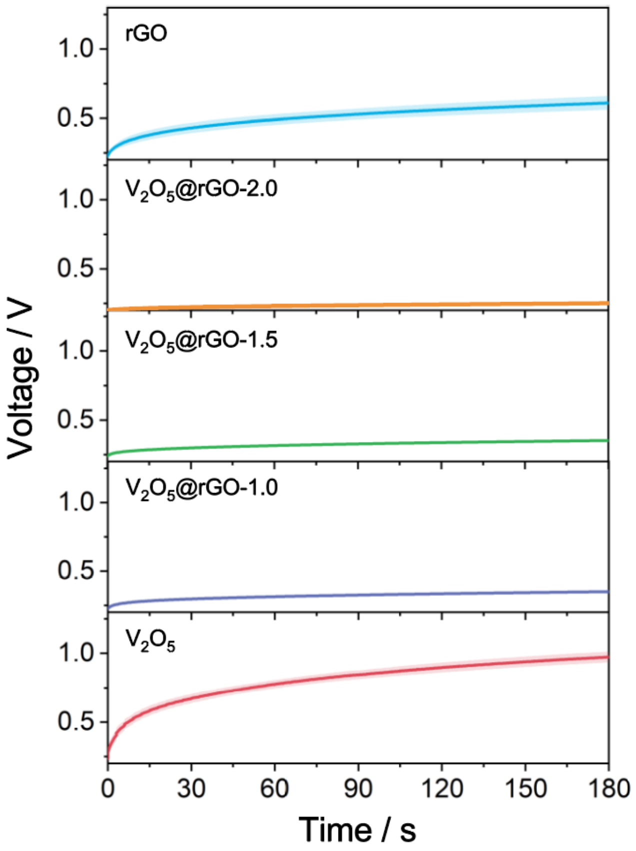

Supplementary Fig. 15 Self-charging curves of H-type ZTECs.

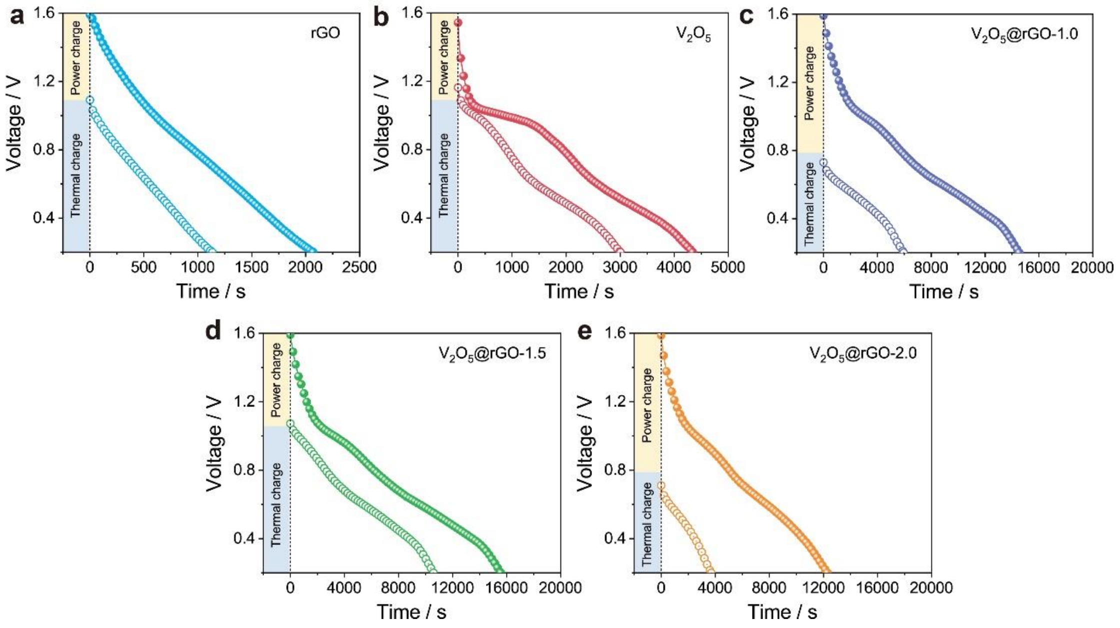

Supplementary Fig. 16 The discharge curves of ZTEC with various cathodes recorded after thermal charge and hybrid charge. a rGO. **b** V_2O_5. **c** V_2O_5@rGO-1.0. **d** V_2O_5@rGO-1.5. **e** V_2O_5@rGO-2.0.

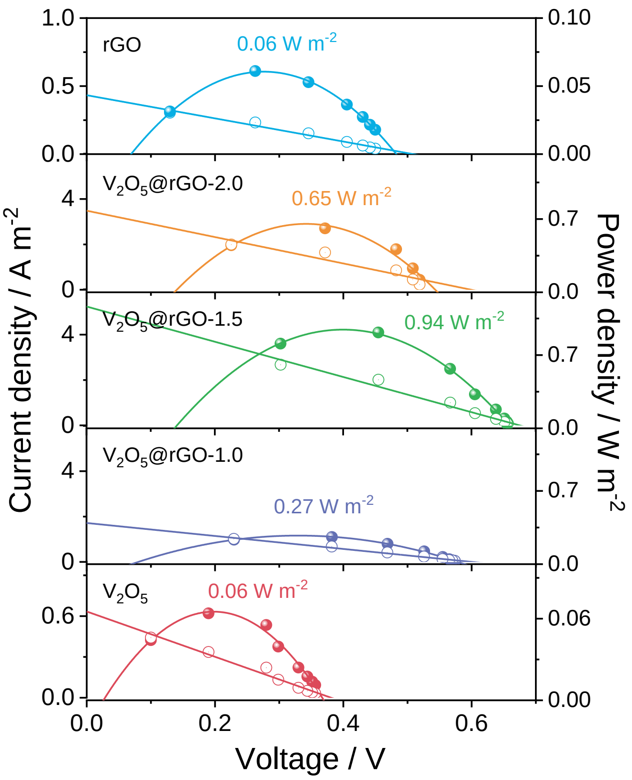

Supplementary Fig. 17 Current densities and power densities under different thermal-induced voltage of H-type ZTECs.

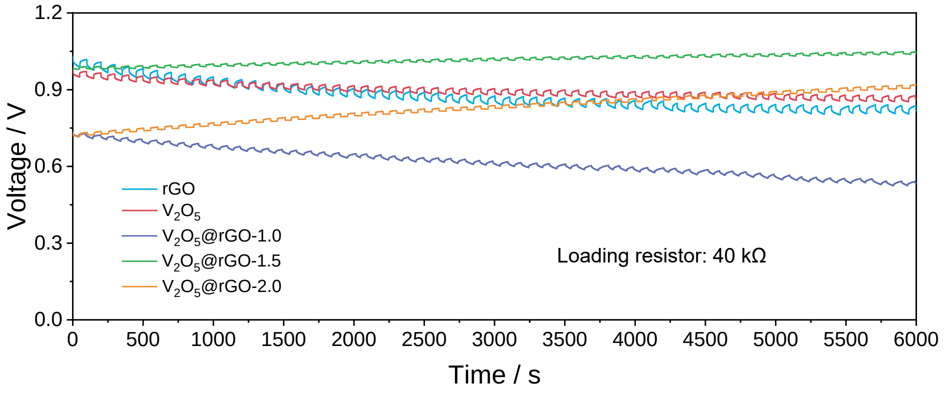

Supplementary Fig. 18 Thermal stability with loading a resistance of 40 kΩ of H-type ZTECs.

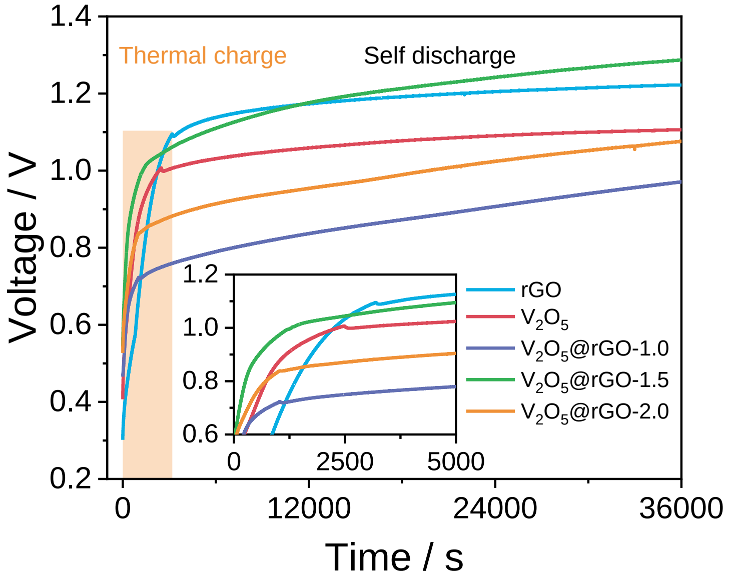

Supplementary Fig. 19 The self-discharge curves obtained after thermal charge.

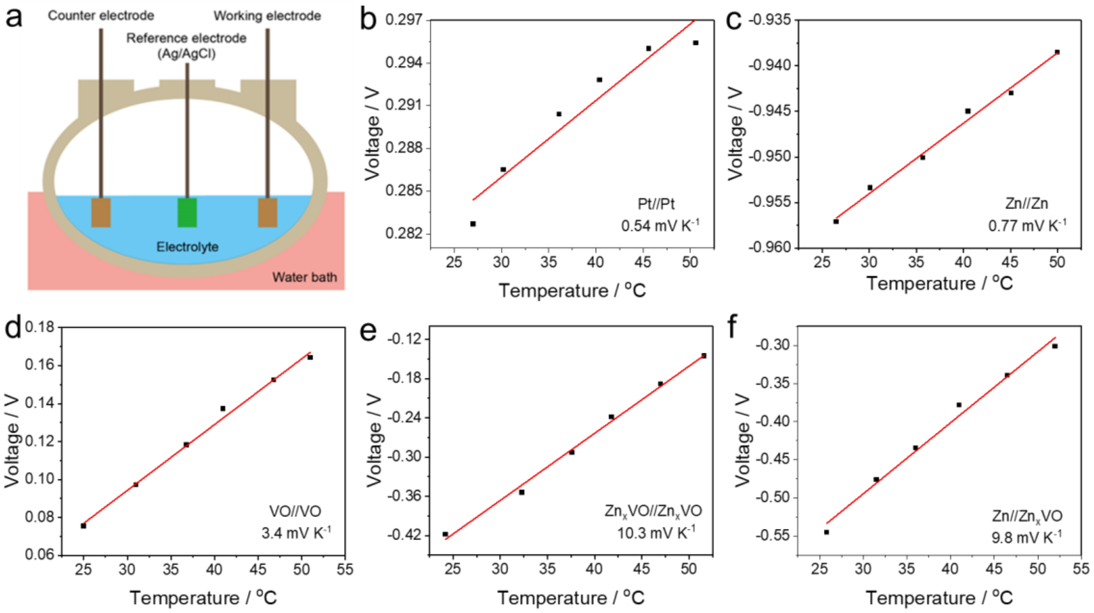

Supplementary Fig. 20 Temperature coefficients of various systems. a Illustration of the three-electrode configuration for isothermal tests. **b-f** Temperature dependence of the open-circuit voltage for various systems.

Supplementary Note 1:

We have tested the related cyclic voltammetry (CV) curves of both Zn-G anode and V_2O_5@rGO-1.5 cathode using three-electrode system (Supplementary Fig. 21). Notably, the Zn-G anode shows the typical plating/stripping behavior, while the V_2O_5@rGO-1.5 cathode presents the reversible redox reaction between vanadium species and zinc ion. According to the CV curves of both electrodes, we can find that the cut-off voltage of Zn-G//V_2O_5@rGO-1.5 could achieve 1.6 V.

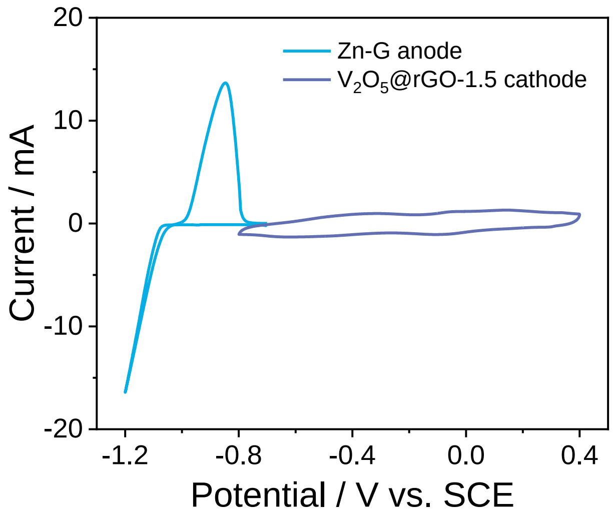

Supplementary Fig. 21 CV curves of Zn-G anode and V_2O_5@rGO-1.5 cathode recorded by three-electrode system with saturated calomel electrode (SCE) as reference electrode.

The electrochemical behaviors of both anode and cathode during the practical cell are further investigated by the potential change of each electrode, respectively. As shown in Supplementary Fig. 22, the Zn^{2+} can be stripped from Zn-G anode and inserted into V_2O_5@rGO-1.5 cathode during the discharge process of Zn-G//V_2O_5@rGO-1.5 full cell. For charge process of Zn-G//V_2O_5@rGO-1.5 full cell, the inserted ions in V_2O_5@rGO-1.5 cathode can be gradually extracted and subsequently plated in Zn-G anode. Based on these processes, the low-grade heat can be converted into electricity and stored in electrodes during the charge part. After discharging by connecting external loads, as-proposed system can be used to further harvest the low-grade heat.

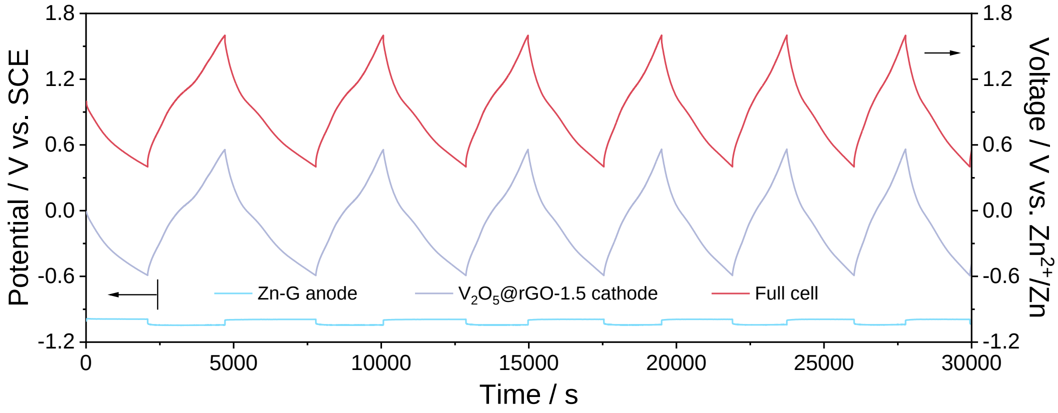

Supplementary Fig. 22 Real-time investigation of voltage for Zn-G anode, V_2O_5@rGO-1.5 cathode and full cell.

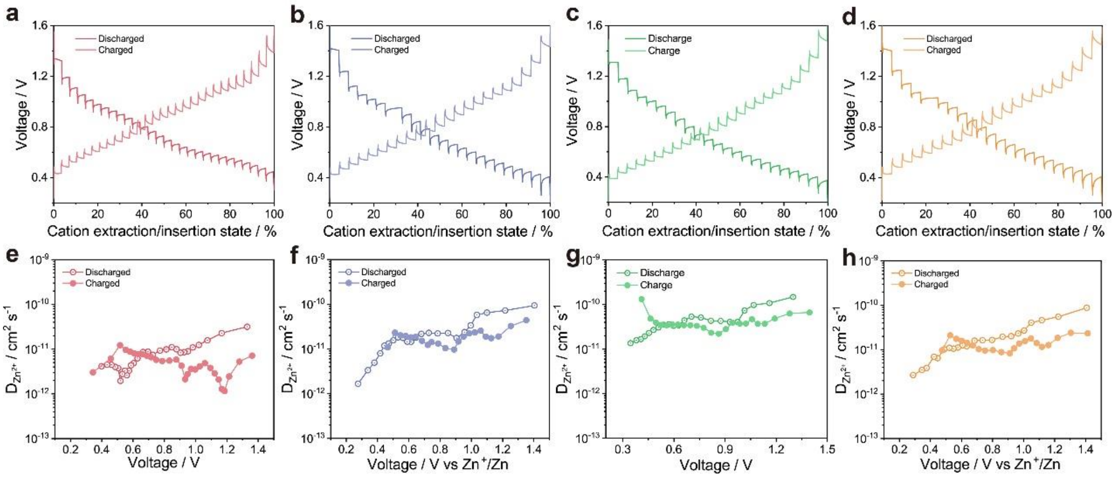

Supplementary Fig. 23 GITT analyses for various electrodes. a,e V_2O_5. **b,f** V_2O_5@rGO-1.0. **c,g** V_2O_5@rGO-1.5. **d,h** V_2O_5@rGO-2.0.

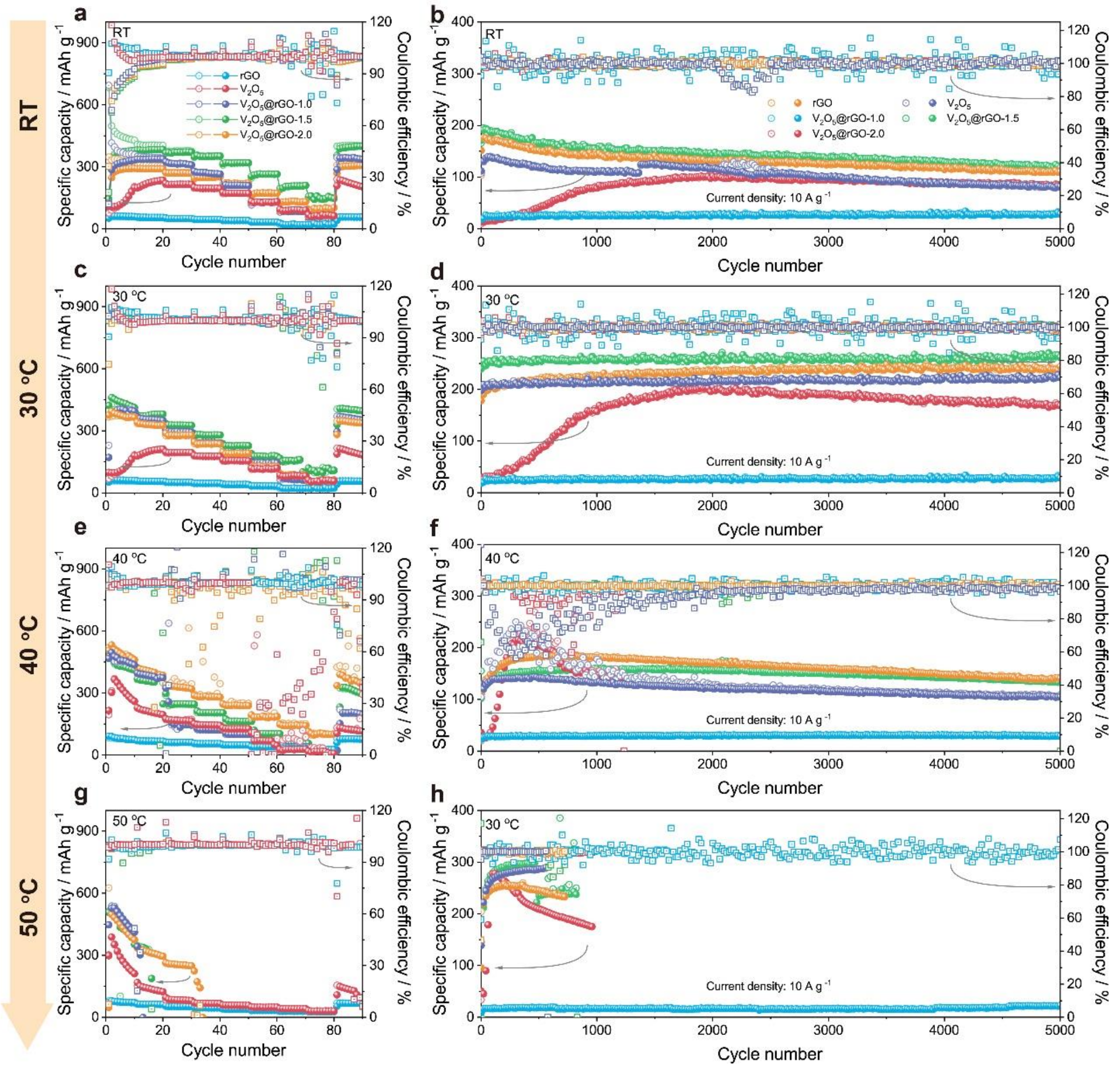

Supplementary Fig. 24 Electrochemical performances of ZIBs under different temperatures. a,b Room temperature. **c,d** 30 ºC. **e,f** 40 ºC. **g,h** 50 ºC.

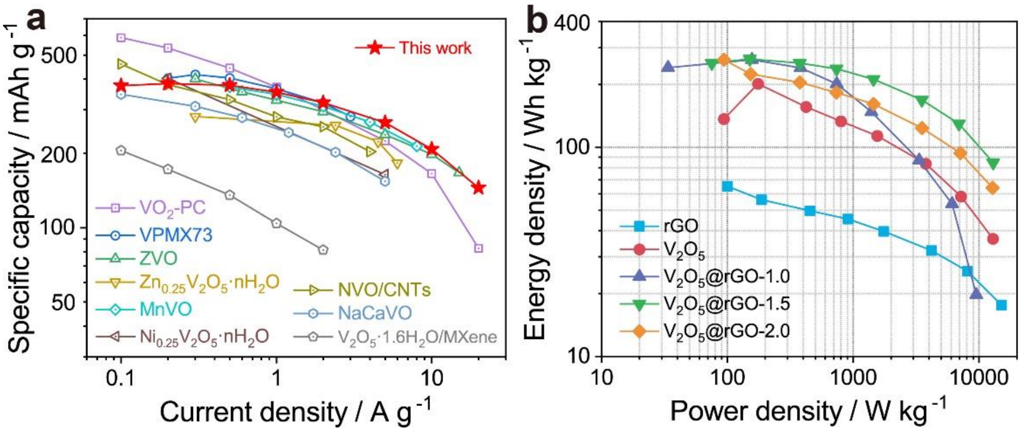

Supplementary Fig. 25 Performances comparation with other reported results. a Specific capacity at different current density. **b** Ragone plots of various devices.

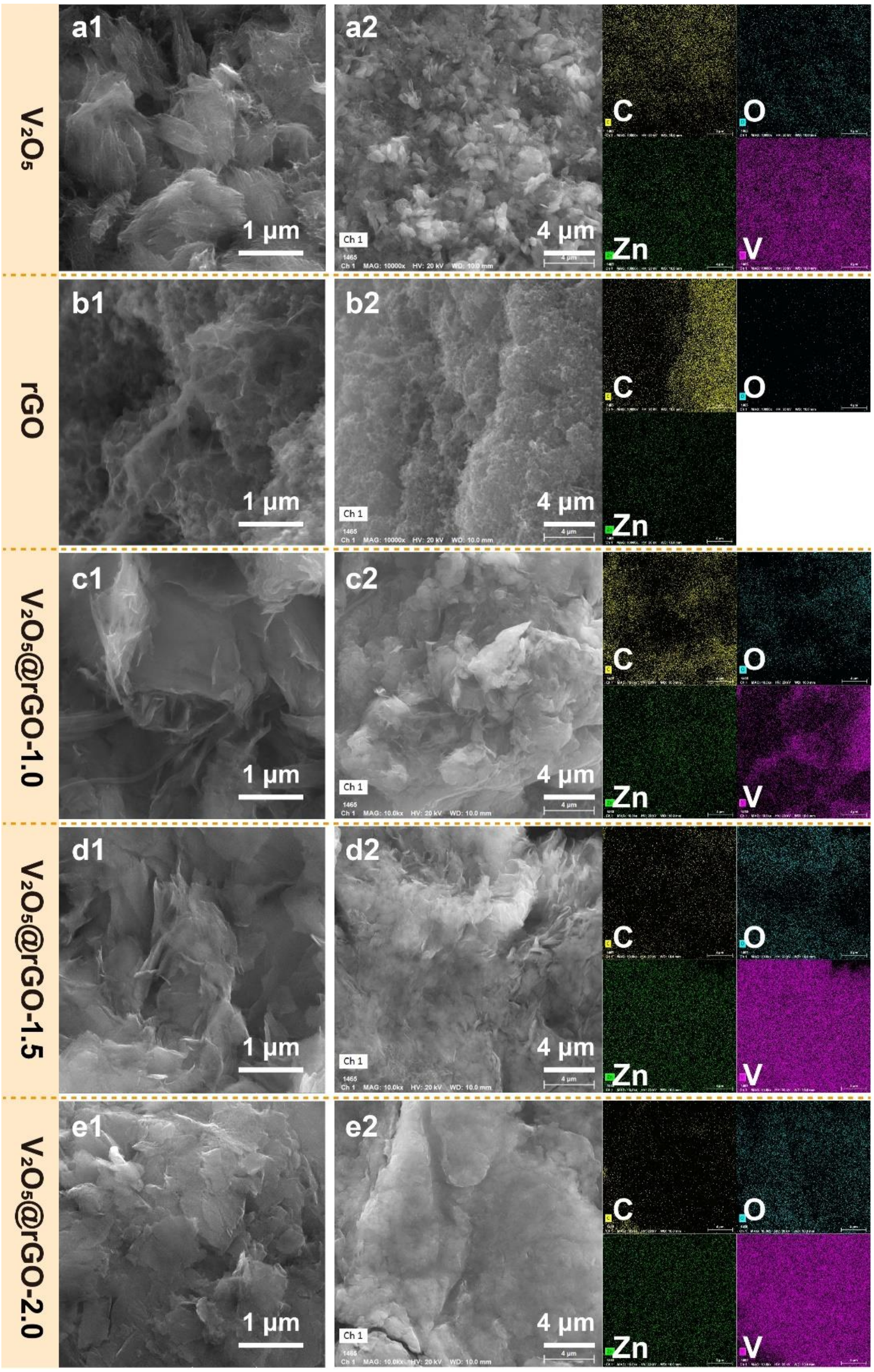

Supplementary Fig. 26 SEM images and EDX mapping images of different electrodes after long-cycling tests. a V_2O_5. **b** rGO. **c** V_2O_5@rGO-1.0. **d** V_2O_5@rGO-1.5. **e** V_2O_5@rGO-2.0.

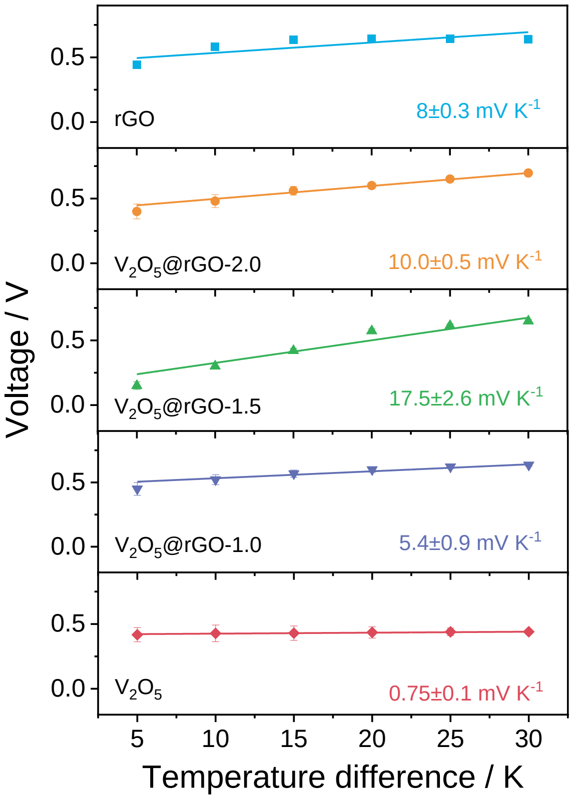

Supplementary Fig. 27 Seebeck coefficient of ZTECs with different electrodes after long-cycling tests.

Supplementary Note 2:

For typical TECs, the thermoelectrochemical conversion highly depends on the redox reaction $A\ ox + n\ e \leftrightarrow B\ red$. Under this case, the equilibrium potential (*E*) of TEC can be descripted as follows according to the Nernst equation:

$$E = E^0 + \frac{RT}{nF}\ln\frac{(\alpha_{ox})^A}{(\alpha_{red})^B} \quad \text{(S1)}$$

where E^0 is the standard potential, *R* is the ideal gas constant, *T* is the temperature of electrode, *n* is the number of electrons transferred in the electrochemical reaction, *F* is the Faraday constant. The *A* and *B* are the coefficients in the redox reaction. α is the activity of the oxidation (*ox*) or reduction species (*red*), which can be defined as:

$$\alpha = \gamma C \quad \text{(S2)}$$

where γ and *C* represent the activity coefficient and concentration.

By combing equation S1 and S2, the equation S1 can be rewritten as:

$$E = E^0 + \frac{RT}{nF}\left[\ln\frac{(\gamma_{ox})^A}{(\gamma_{red})^B} + \ln\frac{(C_{ox})^A}{(C_{red})^B}\right] \quad \text{(S3)}$$

Moreover, the thermopower of a redox couple can be defined as:

$$S_{TGC} = -\frac{E_H - E_C}{T_H - T_C} \quad \text{(S4)}$$

Inspired by the discussion from literatures (*Science, 2020, 368, 1091-1098; Sci. Adv., 2022, 8, eabl5318*), such equation can be further expanded as:

$$S_{TGC} = -\frac{{E_H}^0 - {E_C}^0}{T_H - T_C} - \frac{R}{nF\Delta T}\left[T_H\ln\frac{(\gamma_{ox,H})^A}{(\gamma_{red,H})^B} + T_C\ln\frac{(\gamma_{ox,C})^A}{(\gamma_{red,C})^B}\right] - \frac{R}{nF\Delta T}\left[T_H\ln\frac{(C_{ox,H})^A}{(C_{red,H})^B} + T_C\ln\frac{(C_{ox,C})^A}{(C_{red,C})^B}\right] \quad \text{(S5)}$$

Here, the temperature coefficient (α_R) can be determined by electrochemistry and defined as equation S6:

$$\alpha_R = \frac{{E_H}^0 - {E_C}^0}{T_H - T_C} \quad \text{(S6)}$$

Thus, the equation S5 can be modified as follows:

$$S_{TGC} = -\alpha_R - \frac{R}{nF\Delta T}\left[T_H\ln\frac{(\gamma_{ox,H})^A}{(\gamma_{red,H})^B} + T_C\ln\frac{(\gamma_{ox,C})^A}{(\gamma_{red,C})^B}\right] - \frac{R}{nF\Delta T}\left[T_H\ln\frac{(C_{ox,H})^A}{(C_{red,H})^B} + \right.$$

$$T_C \ln \frac{(C_{ox,C})^A}{(C_{red,C})^B}\Bigg] \quad \text{(S7)}$$

As found, it is a good method to distinguish the contribution of redox species on the thermopower or Seebeck coefficient by the polarization potential of redox peaks in CV curves for typical TECs. It should be pointed out that here mentioned TECs can not exhibit the change of ions concentration. However, due to the exhibition of redox reactions on both electrodes in cold and hot sides, the concentration change of redox species should not be ignored in our ZTCCs. This limitation also clearly shows the difference in typical TECs and our proposed ZTCCs.

We also have tested the related CV curves using H-type cell and coin-type cell, respectively. As summarized in Supplementary Fig. 28, the area enclosed by CV curves of various electrodes enlargers with the increase of adopted temperature difference between anode and cathode, resulting from the completely infiltration of active electrode materials by electrolyte. Notably, the potential values of redox reactions in V_2O_5 and V_2O_5@rGO-x electrodes display slight change. With the increase of the temperature differences, the polarization between oxidized peaks and reduced peaks increases. This phenomenon may be caused by the dendrite growth and byproducts formation on Zn-G anode or the increase of concentration polarization of system.

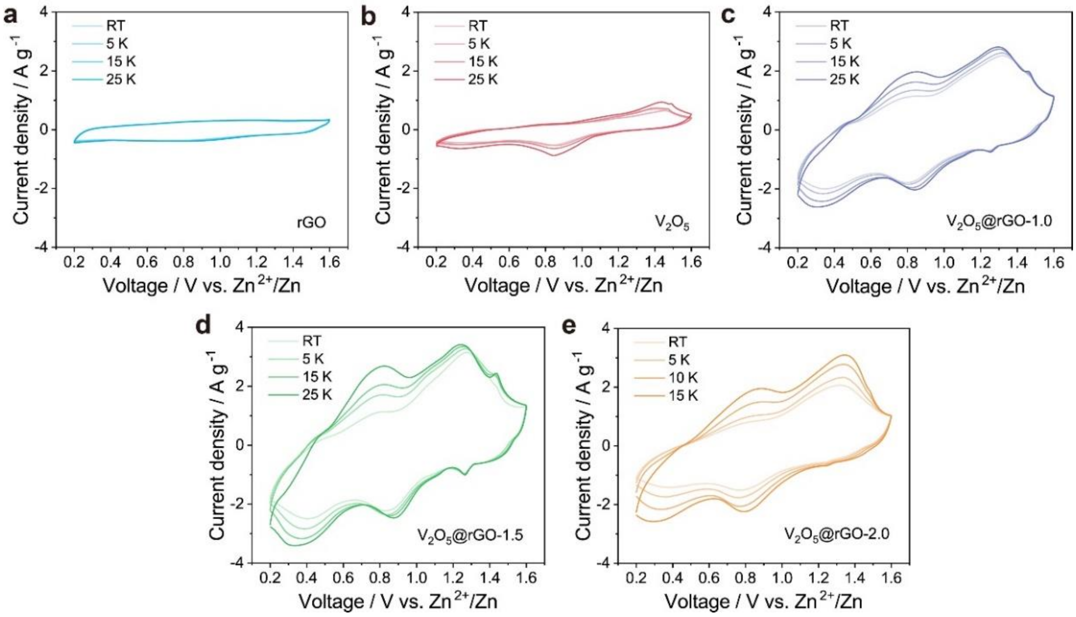

Supplementary Fig. 28 CV curves of H-type ZTECs under various temperature differences. a rGO. **b** V_2O_5. **c** V_2O_5@rGO-1.0. **d** V_2O_5@rGO-1.5. **e** V_2O_5@rGO-2.0.

Moreover, we have further measured the temperature-dependent CV curves of each electrode by using coin-type cells. Similar to the results obtained by H-type cells, the area enclosed by CV curves of various electrodes enlargers with the increase of adopted temperature (Supplementary Fig. 29), which mainly caused by the infiltration of electrolyte ions in cathodes. However, the polarization of redox peaks shows a decrease trend with the increase of temperature. This phenomenon is significantly different with that from H-type cell, and can be attributed to the reduced concentration polarization by rising the temperature of whole cell.

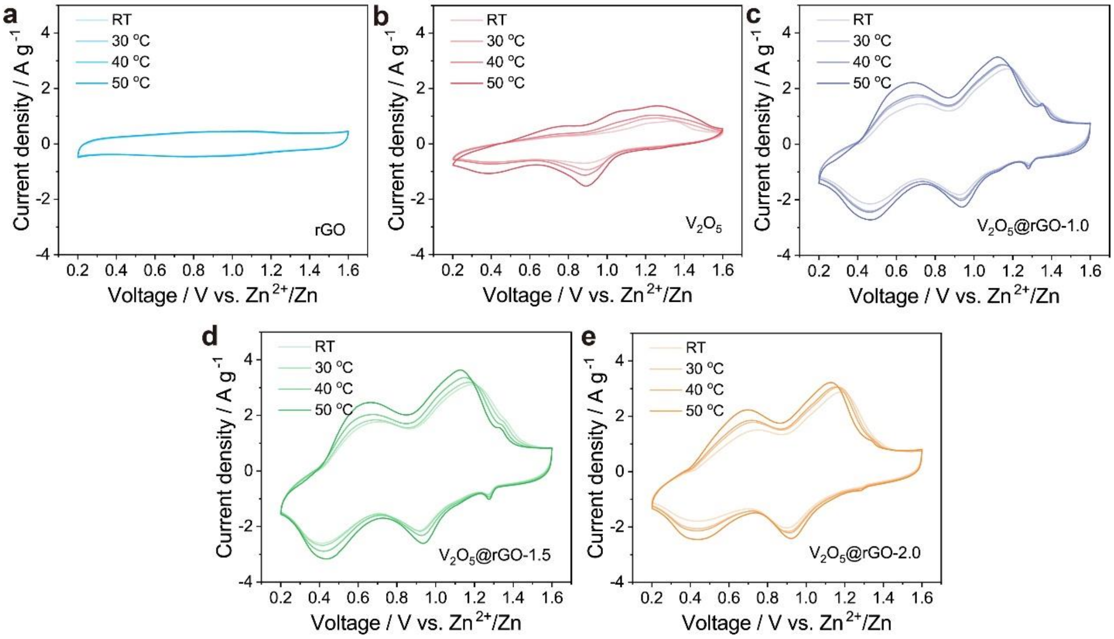

Supplementary Fig. 29 CV curves of coin-type cells under different temperature from RT to 50 ºC. a rGO. **b** V_2O_5. **c** V_2O_5@rGO-1.0. **d** V_2O_5@rGO-1.5. **e** V_2O_5@rGO-2.0.

Overall, the polarization of redox reactions between V_2O_5 and Zn can be significantly optimized by adding moderate rGO. At first, the crystalline structure of orthorhombic V_2O_5 has converted to orthorhombic $V_2O_5·1.6H_2O$ with relatively large interlayer space (~1.1 nm). Secondly, the electron conductivity of pristine V_2O_5 has been enhanced from $1.4×10^{-5}$ to 1.2 S cm^{-1}. Thirdly, the zinc ion diffusion coefficient of V_2O_5 has been improved from $6.7×10^{-12}$ to $4.19×10^{-11}$ cm^2 S^{-1}. According to the significant enhancement of kinetics, high thermoelectrochemical performances as well as good energy storage behaviors can be realized.

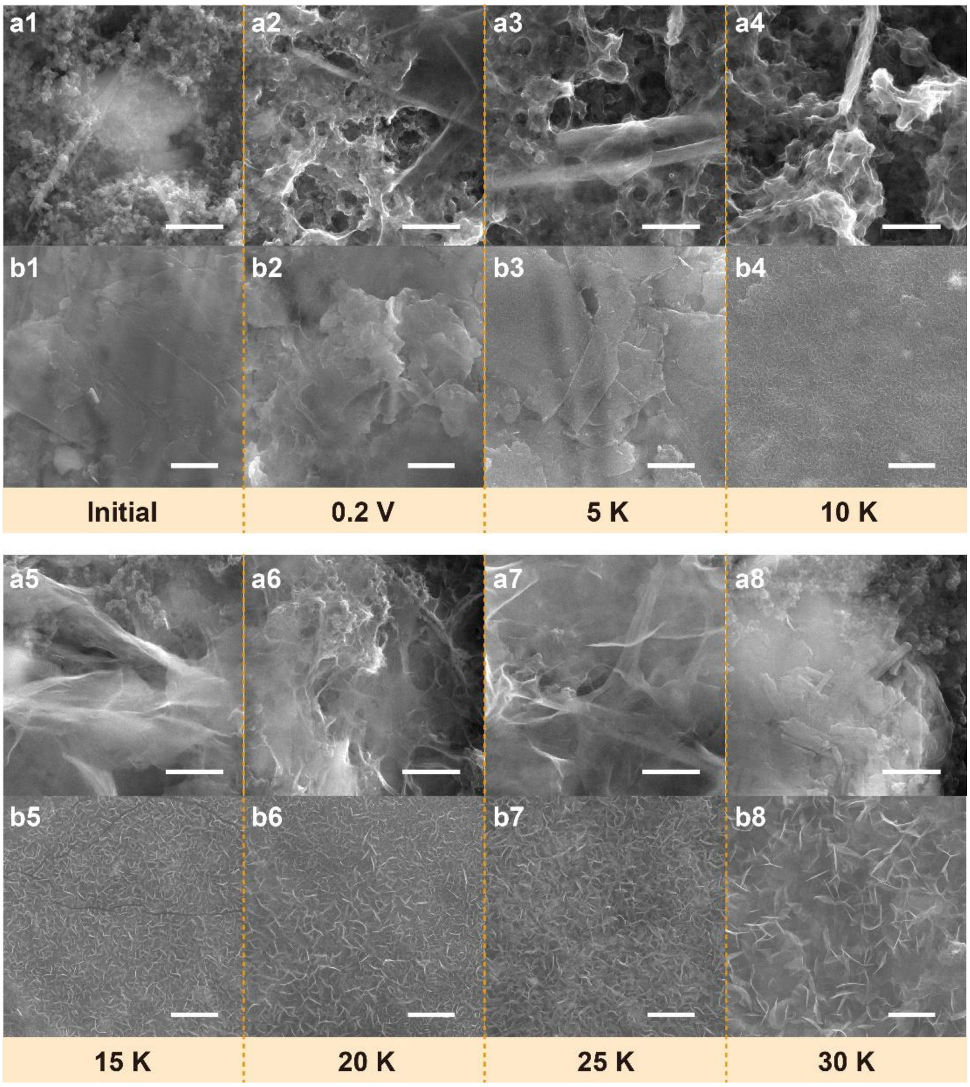

Supplementary Fig. 30 Morphology changes of electrodes during thermoelectrochemical test. a1-a8 V_2O_5@rGO-1.5 cathode and **b1-b8** Zn-G anode under various states.

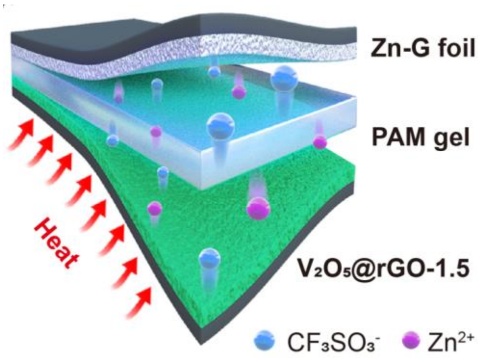

Supplementary Fig. 31 Schematic illustration of the wearable ZTECs.

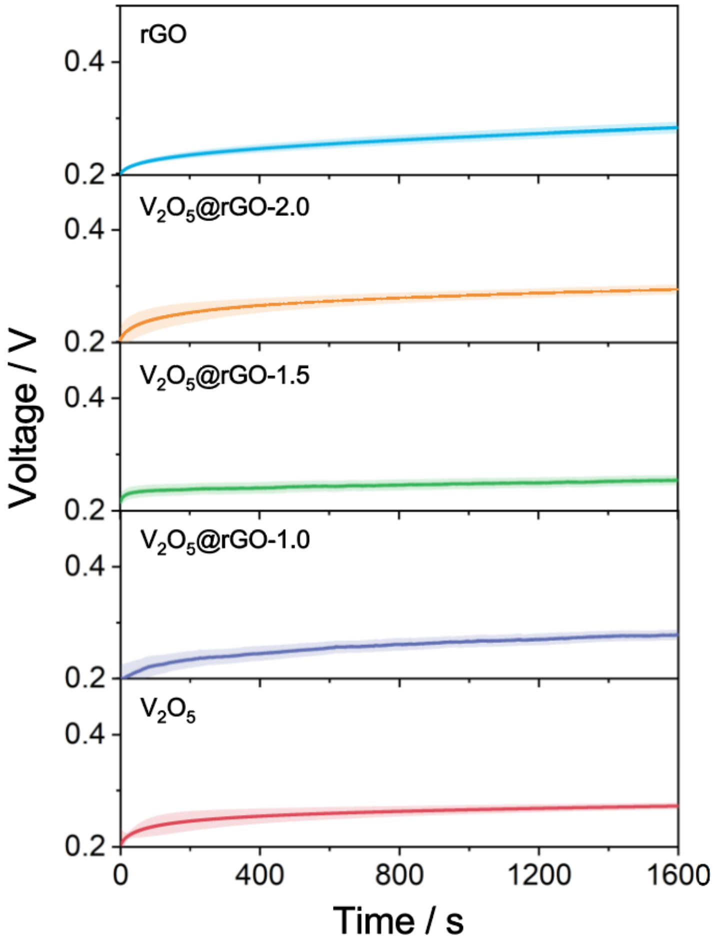

Supplementary Fig. 32 Self-charging curves of quasi-solid-state ZTECs.

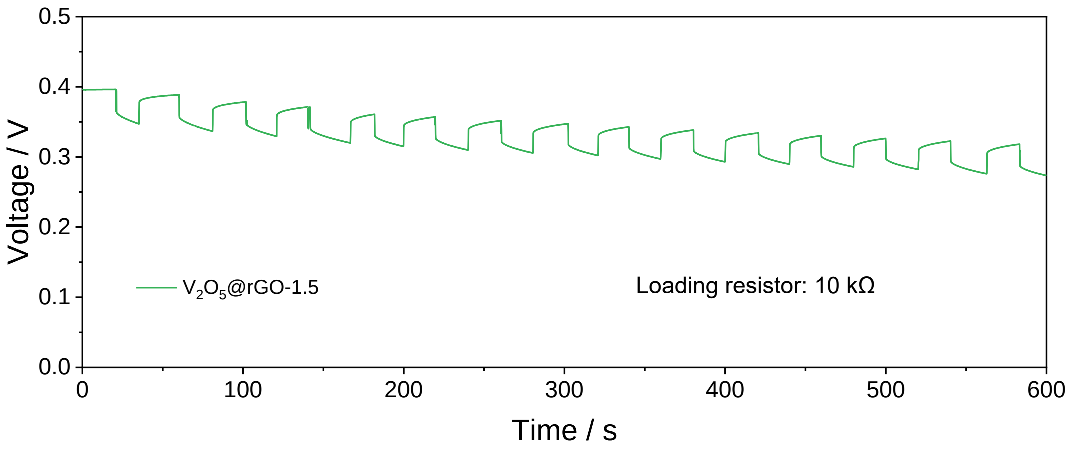

Supplementary Fig. 33 Thermal stability of solid-state ZTCC after 7 days with a loading resistance of 10 kΩ.

Supplementary Table 1. Thermoelectrochemical performances comparison of V_2O_5@rGO-1.5 based solid-state ZTEC with other systems.

System	ΔT^a	$\lvert S \rvert^b$	$P_{max}/(A\times\Delta T^2)^c$	Ref.
Zn-G//V_2O_5@rGO-1.5	8	11.9±1	1.9	**This work**
PAAm based TC	10	~3	0.66	*Adv. Energy Mater.* **12**, 2201542 (2022)
UGdmCl based TGC	10	4.2	1.1	*Nat. Commun.* **9**, 5146 (2018)
3D Au/Cu based i-TE	10	17	0.16	*Adv. Energy Mater.* **12**, 2103666 (2022)
Au/Cu based i-TE	~10	12.7	0.66	*Science* **368**, 1091-1098 (2020)
i-PVA based TC	40	-	0.22	*ACS Nano* **16**, 8347-8357 (2022)
BST/$FeCN^{3-/4-}$ based TC	30.7	4.11	0.96	*Nano Energy* **103**, 107826 (2022)
BC-based TEC	10	~4.5	0.024	*Chem. Eng. J.* **433**, 134550 (2022)

[a] Temperature difference (K).

[b] Seebeck coefficient (mV K^{-1}).

[c] Normalized power density (mW m^{-2} K^{-2}).
